# Immunization programmes and notifications of vital events

**DOI:** 10.2471/BLT.18.210807

**Published:** 2019-01-28

**Authors:** Gustavo Corrêa, Philippe Verstraete, Riswana Soundardjee, Manjari Shankar, Colin Paterson, Lee Hampton, Debra Jackson, Maria Muniz, Remy Mwamba, Kristen Wenz, Martin W Bratschi, Carla AbouZahr, Hope Johnson

**Affiliations:** a Gavi, the Vaccine Alliance, Global Health Campus, Chemin du Pommier 40, 1218 Grand-Saconnex Geneva, Switzerland.; b Health section, United Nations Children’s Fund, New York, United States of America (USA).; c Child Protection section, United Nations Children’s Fund, New York, USA.; d Vital Strategies, New York, USA.; e Bloomberg Data for Health Initiative, University of Melbourne, Melbourne, Australia.

In many low- and middle-income countries, civil registration and vital statistics systems rely on passive reporting, with families expected to attend local registry offices to register vital events, such as births and deaths. However, these families may face physical, cultural, legal, socioeconomic or other barriers that impede their access to registration and reduce the performance of civil registration and vital statistics systems.^1^

**Fig. 1:**
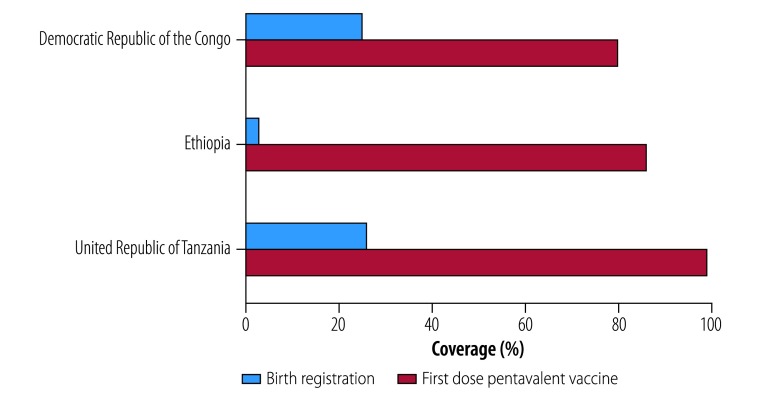
Comparison of birth registration and vaccine coverage rates in selected countries

Globally, one in every four children younger than five years has not been registered at birth.^2^ In sub-Saharan Africa, approximately half of children in this age category have unregistered births, one third of them living in just three countries: the Democratic Republic of the Congo, Ethiopia and United Republic of Tanzania.^3^ In all three countries, immunization, as measured by estimated coverage with the first dose of the pentavalent vaccine for diphtheria, tetanus, pertussis, hepatitis B and *haemophilus influenzae* type B, reaches at least three times more children in the first two months of life than birth registration (Fig. 1).^1^ The reach of vaccination highlights the potential for health systems to reach children in the first months of life in many low- and middle-income countries. Immunization programmes are particularly well placed to help with birth registration efforts in these countries. Vaccination sessions are often the first interaction between children and a government-supported system, and a potential opportunity for notification and official registration of vital events. Immunization is often provided widely throughout countries through both health facility-based services and community outreach.

Data source: WHO-UNICEF Estimates of DTP1 coverage. UNICEF Data: Child Protection: Birth Registration, 2017.

Note: Pentavalent vaccine for: diphtheria, tetanus, pertussis, hepatitis B and *haemophilus influenzae* type B

With adequate capacity building and resources, heath workers can promote, educate and mobilize communities for civil registration and vital statistics system strengthening, and report identified vital events to civil registration authorities.

This approach has been successfully piloted across different countries. In Bangladesh, for example, since most births happen at home, the government decided to link birth registration with the successful immunization programme. Birth registration is now part of regular training of health workers, who can report new births to registrars through standard forms. In 2017, a link between civil registration and vital statistics and the immunization programme was created by adding a field for the birth registration number to the immunization card, ensuring that agents performing immunization sessions know the registration status of children, and empowering them to act as required. This initiative contributed to double birth registration rates in the pilot area (AbouZahr C & Bratschi M, Bloomberg Data for Health Initiative and Vital Strategies, personal communication, 14 August 2018). In Liberia, the United Nations Children’s Fund (UNICEF) supports capacity building and policy development for health workers and civil registration staff. Civil registrars are trained and operate jointly with immunization providers. In 2016, this initiative played a key role in the birth registration of over 140 000 children, also doubling results from the previous year.^4^

Community health workers involved in immunization programmes may also play a crucial role, especially in hard-to-reach areas and for vulnerable populations. In Mali, for example, community health workers providing health-promotion services to vulnerable populations and reporting vital events as part of their formal job description were able to collect good-quality data on routine vital events in a defined geographic area.^5^

Information sharing and triangulation between immunization programmes and civil registration and vital statistics databases at the local and national level could reveal unregistered children and potentially identify duplicate records. Cross-sectoral cooperation between health programmes, statistics offices, civil registration programmes, local governments, home affairs and other sectors, could then result in a marked increase in the quantity and quality of civil registration and vital statistics data to inform policy and practice.

With more complete and reliable data, civil registration and vital statistics systems will benefit immunization programmes in multiple ways. One of the most relevant contributions is the provision of population estimates to target vaccination efforts and monitor the reach of immunization programmes.

Robust population estimates based on complete and reliable civil registration and vital statistics data are critical to enable countries to provide quality immunization services; ensure that vaccines are available, procured and distributed where they are needed; reach vulnerable and underserved populations; and identify children and ensure they receive complete and timely vaccination according to schedule. However, population estimates are inaccurate in many countries.

Often, population estimates are based on extrapolation from censuses, adjusted using projected growth rates based on mathematical models. Furthermore, in some countries censuses are not conducted on a regular basis. In the Democratic Republic of the Congo and Eritrea, for example, the most recent censuses date is from 1984.^6^ The lack of reliable population hampers planning for immunization services and requisition of an adequate number of vaccines doses. Even in countries where censuses are regularly conducted, population dynamics, including migration, refugee crises and rapid urbanization rates make population projections highly inaccurate, especially at the local level. Urban slums are particularly challenging given their frequently rapid growth and the political complexities of registering populations and providing services.

Additionally, strong civil registration and vital statistics systems can also provide accurate mortality data that contribute to identifying deaths caused by vaccine-preventable diseases. This may facilitate the detection of and response to outbreaks, identification of populations insufficiently protected from vaccine-preventable diseases, planning of vaccination campaigns, targeting of immunization resources and the introduction of new vaccines against high-burden diseases.

Finally, the absence of birth certificates makes it difficult to ensure timely vaccination. As countries usually follow the World Health Organization’s (WHO) recommended vaccination schedule based on a child’s age, lack of birth registration may be an important barrier for adequate and age-appropriate vaccination. This can negatively impact vaccine coverage and health and reduce the cost–effectiveness of immunization programmes.^7^

Given that both birth and death registration levels have seen little progress in the poorest countries over the past 30 years,^8^ a change in the global civil registration and vital statistics paradigm is needed, including an increased focus on coordination across programmes and sectors. Strengthening civil registration and vital statistics systems is a priority and a key concern for international organizations and is included in sustainable development goals (SDGs) 16 and 17.

Gavi recommends and supports the strengthening of civil registration and vital statistics systems through its available funding mechanisms, such as health system strengthening grants and targeted country assistance through technical partners. In addition, Gavi supports partners to conduct case studies capturing lessons learnt in civil registration and vital statistics strengthening in countries like Cameroon, Democratic Republic of the Congo, Ethiopia, Kenya, Liberia, Nepal and Uganda. However, much more can be done. 

Global institutions such as UNICEF, WHO and the World Bank have key roles to play through initiatives, such as the Open Learning Campus, which provides a civil registration and vital statistics e-learning course for management and analysis of civil registration and vital statistics data in low- and middle-income countries.

New digital technologies are also promising areas for civil registration and vital statistics system strengthening, with the potential of bringing long-term benefits in low-resource settings at an affordable price. Data safety will be extremely relevant for such technologies to be widely adopted. Ensuring that personal data is safeguarded in many countries, in terms of system design, data protection and privacy legislation is still an ongoing process.^9^ Once this barrier is overcome, interconnecting immunization digital technologies like electronic immunization registers with civil registration and vital statistics systems could potentially be transformative in the short-term. Opportunities may also arise where digital civil registration and vital statistics initiatives are being piloted. This was demonstrated through a successful interconnectedness initiative in rural Malawi, which linked health passports (patient-kept medical records) to the electronic village register.^10^ In the United Republic of Tanzania, for example, a recent initiative successfully tested an electronic decentralized birth registration system based on mobile short message service technology, but with significant challenges to scale.^11^ Linking the birth registration system to immunization systems could potentially provide a platform for broader implementation.

The global community needs to have a bold vision for civil registration and vital statistics strengthening, and be ready to identify and scale up successful pilots and promising interventions. To this end, Gavi launched a new initiative in 2016: Innovation for Uptake, Scale and Equity in Immunisation (INFUSE). This initiative aims to identify and scale tested innovations with the potential to improve vaccine delivery, and welcomes proposals for digital technologies that interconnect immunization programmes and civil registration and vital statistics systems.

Coordination of civil registration and vital statistics strengthening efforts is critical. A broad-based international coalition, including state and non-state actors, development partners, civil society, the private sector and academic institutions is needed to effectively respond to countries’ needs and priorities.^12^ The ID4D and the ID2020 Alliance are committed to bringing together governments, civil society, international organizations and the private sector to efficiently implement digital identity technologies at scale.

Partnerships offering support to governments and partners at the global and regional levels and aligning partners’ data investments around a common civil registration and vital statistics agenda, such as the Global and Regional CRVS Groups and the Health Data Collaborative, can also play an important role, although more financial, technical and political commitment will be needed in the near future.

Considerable progress towards the SDG target of providing legal identity for all can be achieved, with the involvement of national governments and in-country partners. Capitalizing on the experience of international partners and welcoming new actors to accelerate civil registration and vital statistics system strengthening will also be necessary. We encourage countries and partners to apply for civil registration and vital statistics strengthening and technical assistance through Gavi’s funding mechanisms.
